# *ZNF367* Inhibits Cancer Progression and Is Targeted by miR-195

**DOI:** 10.1371/journal.pone.0101423

**Published:** 2014-07-21

**Authors:** Meenu Jain, Lisa Zhang, Myriem Boufraqech, Yi Liu-Chittenden, Kimberly Bussey, Michael J. Demeure, Xiaolin Wu, Ling Su, Karel Pacak, Constantine A. Stratakis, Electron Kebebew

**Affiliations:** 1 Endocrine Oncology Branch, Center for Cancer Research, National Cancer Institute, National Institutes of Health, Bethesda, Maryland, United States of America; 2 Translational Genomic Research Institute, Phoenix, Arizona, United States of America; 3 Laboratory of Molecular Technology, SAIC-Frederick, Inc., Frederick National Laboratory for Cancer Research, Frederick, Maryland, United States of America; 4 Section on Medical Neuroendocrinology, Eunice Kennedy Shriver National Institute of Child Health, National Institutes of Health, Bethesda, Maryland, United States of America; 5 Section on Endocrinology and Genetics, Eunice Kennedy Shriver National Institute of Child Health, National Institutes of Health, Bethesda, Maryland, United States of America; University of Alabama at Birmingham, United States of America

## Abstract

**Background:**

Several members of the zinc finger protein family have been recently shown to have a role in cancer initiation and progression. Zinc finger protein 367 (*ZNF367*) is a member of the zinc finger protein family and is expressed in embryonic or fetal erythroid tissue but is absent in normal adult tissue.

**Methodology/Principal Findings:**

We show that *ZNF367* is overexpressed in adrenocortical carcinoma, malignant pheochromocytoma/paraganglioma and thyroid cancer as compared to normal tissue and benign tumors. Using both functional knockdown and ectopic overexpression in multiple cell lines, we show that *ZNF367* inhibits cellular proliferation, invasion, migration, and adhesion to extracellular proteins *in vitro* and *in vivo*. Integrated gene and microRNA expression analyses showed an inverse correlation between *ZNF367* and miR-195 expression. Luciferase assays demonstrated that miR-195 directly regulates *ZNF367* expression and that miR-195 regulates cellular invasion. Moreover, integrin alpha 3 (*ITGA3*) expression was regulated by *ZNF367*.

**Conclusions/Significance:**

Our findings taken together suggest that *ZNF367* regulates cancer progression.

## Introduction

Our knowledge of the biologic events that inhibit or promote the metastatic spread of endocrine cancers locally and to distant sites is limited [1]. Understanding the molecular events involved in cancer progression has important implications for identifying targets for therapeutic benefit. Genomic, genetic, and epigenetic approaches have been used to identify changes in genes/pathways, transcription factors, or gene signatures by comparing normal, primary tumors, and/or metastasis. However, these analyses have not often distinguished between promoters, inhibitors, or passenger markers in cancer initiation and progression, and rarely define a specific mechanism for dysregulated gene expression.

Endocrine cancers are a diverse group of malignancies that exhibit the full spectrum of biologic behavior of malignant tumors: indolent growth to rapidly progressive cancers with poor survival. Thus, endocrine cancers represent an excellent model for studying the molecular factors that influence cancer initiation and progression. Identifying genetic changes common to these diversely behaving endocrine tumor types has been shown to play an important role in many types of human cancers. For example, *BRAF* mutations are highly prevalent in thyroid cancer and other cancers, and are associated with more aggressive disease in thyroid cancer [2], [3]. *SDHB* mutations were initially identified in familial paraganglioma, but were later found to be prevalent in kidney cancers and gastrointestinal stromal tumors, and recently pituitary tumors [2], [4], [5]. Thus, discovering the molecular changes associated with endocrine cancer initiation and/or progression is likely to play an important role in a broad group of human malignancies.

In this study, we determined the mechanism of gene expression regulation and function of *ZNF367* in a variety of endocrine cancers (papillary thyroid cancer, adrenocortical carcinoma, pheochromocytoma/paraganglioma). *ZNF367* (also known as *ZFF29* and *CDC14B*) is a member of the zinc finger protein family, with a unique Cys2His2 zinc finger motif, is expressed in embryonic or fetal erythroid tissue, and is absent in other normal adult tissue [6], [7]. Recently, several zinc finger proteins have been found to be dysregulated in cancer, to function as tumor suppressors/promoters, and to cause resistance to chemotherapy [8]–[10]. However, the role of *ZNF367* in cancer has not been investigated. Here, we report that *ZNF367* is overexpressed in a variety of endocrine cancers and that it inhibits *in vitro* and *in vivo* growth, cellular invasion, migration, and adhesion. Moreover, *ZNF367* overexpression is associated with the loss of miR-195 expression, which directly targets *ZNF367*. Lastly, *ITGA3* is decreased with *ZNF367* overexpression, establishing a miR-195-*ZNF367*-*ITGA3* axis that functions to inhibit cancer progression.

## Methods

### Tissue samples and animal studies

Tissue samples were procured on an Institutional Review Board–approved clinical protocol after obtaining written informed consent (ClinicalTrials.gov Identifier: NCT01005654). The tissue samples were collected at the time of surgery, and they were immediately snap frozen and stored at −80°C. One-hundred-and-five human adrenocortical (19 normal adrenal cortex, 79 cortical adenomas, 7 adrenocortical carcinomas), 47 thyroid tissue (8 normal, 39 papillary thyroid cancer), and 68 (19 normal adrenal medulla, 28 benign, 21 malignant pheochromocytoma/paraganglioma) tissue samples were used in this study. All diagnosis was confirmed by an endocrine pathologist, and tumor samples were confirmed to contain ≥ 80% tumor cells/nuclei. The diagnosis of adrenocortical carcinoma and malignant pheochromocytoma/paraganglioma was based on the presence of gross local invasion and/or distant metastasis and a Weiss score of > 3 for adrenocortical carcinoma. Normal adrenal cortex and medulla were collected from healthy organ donors using laser capture microdissection under an Institutional Review Board–approved protocol. Two publicly available datasets of genome-wide gene expression analysis in adrenocortical tumor samples were used to evaluate *ZNF367* mRNA expression (http://www.ebi.ac.uk/arrayexpress/experiments/E-GEOD-10927 and http://www.ebi.ac.uk/arrayexpress/experiments/E-TABM-311).

The National Cancer Institute Animal Care and Use Committee approved the protocols for animal care and handling in the present study. Any mouse experiencing significantly abnormal neurological signs, bleeding from any orifice, impaired mobility, rapid weight loss, debilitating diarrhea, rough hair coat, hunched posture, labored breathing, lethargy, persistent recumbence, jaundice, anemia, self-induced trauma, becomes moribund or otherwise becomes unable to obtain food or water, or with a tumor 2 cm or greater in diameter has been immediately euthanized by CO_2_ chamber.

### Cell lines, cell culture, reagents, siRNA, and miR-195 transfection

The SW13 adrenocortical carcinoma cell line (ATCC, Rockville, MD) was grown and maintained in DMEM media supplemented with 1% insulin transferrin selenium (ITS; BD Biosciences, San Jose, CA) and 2.5% Nu-Serum I (BD Biosciences) in a standard humidified incubator at 37°C in a 5% CO_2_ atmosphere. The BD140A adrenocortical carcinoma cell line was kindly provided by Dr. Kimberly Bussey (TGen, Pheonix, Arizona), and it was cultured in RPMI media supplemented with 10% FBS (Invitrogen, Carlsbad, CA), 1% Penicillin-Streptomycin, and 1% L-glutamate. The human papillary thyroid cancer (TPC-1) cell line was maintained in DMEM supplemented with FBS, penicillin (100 U/ml), streptomycin (100 µg/ml), fungizone (250 ng/ml), TSH (10 I.U/L), and insulin (10 µg/ml). Human embryonic kidney (HEK293) cells were maintained in DMEM supplemented with 10% FBS, 1% Pen-strep (Gibco, Grand Island, NY), and 1% L-glutamate (Gibco). All the cell lines were authenticated by short-tandem repeat profiling on October 14, 2012.

ZNF367 siRNAs (s46962, s46963) and negative control siRNAs (AM4613) were used at a final concentration of 80 nM (Applied Biosystems, Foster City, CA). Lipofectamine RNAiMAX (Invitrogen, Carlsbad, CA) was used for cell transfection of siRNAs. Mature miRNA precursor, pre-miR-195, and random sequence pre-miR-negative control (Applied Biosystems) at 5 nM were transfected into SW13 cells using Lipofectamine RNAiMAX (Invitrogen, Carlsbad, CA). For *ZNF367* overexpression in HEK293 cells, cells (8×10^5^ in each 6 well) were transfected with a *ZNF367* cDNA construct or an empty vector (OriGene, Rockville, MD) using the Lipofectamine 2000 transfection reagent (Invitrogen).

### Immunohistochemistry

Formalin-fixed and paraffin-embedded tissue sections were de-paraffinized and then rehydrated using xylene and ethanol. Antigen retrieval was completed using a 10% citrate buffer pH 6.0 (Thermo Scientific, Lafayette, CO) in a pressure cooker at 120°C for 10 minutes. Tissue sections were incubated with 6% hydrogen peroxide to quench endogenous peroxidase activity for 30 minutes (Dako, Carpinteria, CA), followed by incubation with serum for an hour (Dako, Carpinteria, CA). Primary anti-ZNF367 rabbit polyclonal antibody (Sigma, St. Louis, MO; HPA015785) at 5 µg/ml dilution was used overnight at 4°C. Anti-rabbit secondary antibody was used (Dako Envision anti-rabbit, Carpinteria, CA) for 1 hour at room temperature. Sections were developed using 3,3′-diaminobenzidine DAB as the chromogen (Dako, Carpinteria, CA), and they were counterstained with hematoxylin. The sections were dehydrated and mounted with vectamount mounting medium (Vector Laboratories, Burlingame, CA). The immunostaining of ZNF367 was evaluated by light microscopy (Nikon, Tokyo, Japan), and images were scanned at 20X magnification.

### RNA preparation, reverse transcription, and real-time quantitative PCR

RNA was isolated using the TRIzol reagent, according to the manufacturer's instructions (Invitrogen Inc., Carlsbad, CA). Total RNA (200–500 ng) was reverse-transcribed using a High Capacity Reverse Transcription cDNA kit, and cDNA was amplified according to the manufacturer's instructions (Applied Biosystems, Foster City, CA). The PCR primers and probes for *ZNF367* (Hs00400665_m1), *ITGA3* (Hs01076873_m1), and *GAPDH* (Hs_99999905_m1) were obtained from Applied Biosystems.

### Western blot

Lysates were prepared with 1% SDS plus 10 mM Tris [pH 7.5] buffer, and Western blot was performed on 10% SDS-PAGE gel. Primary rabbit polyclonal antibodies, anti-ZNF367 (HPA015785; Sigma, MO) and anti-ITGA3 (Sigma; SAB1100194) were used at 5 µg/ml and 2 µg/ml, respectively, and anti-GAPDH (sc-32233; Santa Cruz Biotechnology Inc., Santa Cruz, CA) was used at 1∶1,000 dilution. Secondary anti-rabbit antibody was used at 1∶5,000 dilution (Cell Signaling, Danvers, MA).

### Cell proliferation

Cells were seeded at a concentration of 2,000 cells in a 96-well plate in six replicates. The CyQUANT™ assay kit (Invitrogen) was used to evaluate the cell number, according to the manufacturer's instructions.

### Clonogenic assay

HEK293 cells (8×10^5^) were transfected with the empty vector or *ZNF367* construct. After 24 hours, cells were trypsinized, re-suspended in the media, and counted. The cells were re-seeded in 6-well plates at 250, 500, and 1,000 cells. After 10 days, media was removed from the wells and washed twice with ice-cold PBS. The colonies were fixed with 4% paraformaldehyde for 20 minutes and stained with 2 ml of crystal violet for 60 minutes on a rocking platform. The plates were washed three times with PBS and air-dried, and the colonies were photographed in FluorChem Imager (San Jose, CA).

### Cell invasion and migration assay

Cellular invasion and migration were assessed using the BD BioCoat Matrigel Invasion Chamber (BD Biosciences) according to the manufacturer's protocol. 1×10^5^ cells were seeded onto the inserts (8-µM pore-sized polycarbonate membranes) with and without a thin layer of Matrigel Basement Membrane Matrix (BD Biosciences) coat. The inserts were placed into bottom wells, with 10% serum-containing culture medium as a chemoattractant. The plates were incubated for 48 hours at 37°C. Cells that invaded the Matrigel matrix or that migrated through the pores without Matrigel matrix to the lower surface of the membrane were fixed and stained with Diff-Quik (Dade Behring, Newark, NJ) and counted under a light microscope in four separate fields with Image J software (NIH, Bethesda, MD).

### Cell adhesion assay

SW13 cells were transfected with *ZNF367* siRNA and the negative control. After 120 hours of transfection, cells were trypsinized, and 1×10^5^ cells were plated in a 48-well plate containing five adhesion molecules (Fibronectin, Collagen I, Collagen IV, Laminin I, and Fibrinogen) and incubated at 37°C for 90 minutes according to the manufacturer's instructions (CytoSelect, Cell Biolabs, Inc., San Diego, CA). The wells were washed twice with PBS, and 200 µl of the staining solution was added and incubated for 10 minutes. The wells were washed twice with deionized water. The wells were air-dried, and 200 µl of the extraction solution was added and incubated for 10 minutes. A total of 150 µL was transferred from each extracted sample to a 96-well plate, and absorbance measured at 560 nm on a SpectraMax M5e microplate reader (Sunnyvale, CA).

### Genome-wide mRNA expression microarray

SW13 cells were transfected with *ZNF367* siRNA and the negative control in triplicate. Following transfection, total RNA was extracted using the RNeasy (Qiagen, Valencia, CA) kit, according to the manufacturer's instructions. cDNA reverse transcription, synthesis, amplification, fragmentation, and terminal labeling of 150 ng total RNA were performed using the GeneChip WT Sense Target Labeling and Control Reagents (Affymetrix, Santa Clara, CA). A total of 25 ng/µL of cDNA was hybridized to the Affymetrix Human Gene 1.0 ST Array GeneChip. Pathway analysis was performed using the DAVID bioinformatics resources (http://david.abcc.ncifcrf.gov, Frederick, MD).

### *In silico* identification of microRNA targeting ZNF367

MiR databases (Target Scan (http://www.targetscan.org) and mirDB (http://mirdb.org/miRDB/) were used to identify microRNA that may target *ZNF367*. Candidate microRNAs predicted to target *ZNF367* were then analyzed to determine if they were differentially expressed in adrenocortical carcioma and papillary thyroid cancer.

### Luciferase assay

Wild-type *ZNF367* 3′UTR was cloned into the GoClone construct (Switchgear Genomics, Menlo Park, CA). The mutant construct of *ZNF367* 3′UTR was obtained by introducing the mutation into the first three nucleotides of the seed region (143–150, GCTGCTA -- CGAGCTA) for miR-195. Wild-type *ZNF367* 3′UTR or mutant *ZNF367* 3′UTR and the empty 3′UTR vector with pre-miR-195 or pre-NC were co-transfected into SW13 cells (Switchgear Genomics). The normalization method for transfection efficiency in luciferase reporter assay was according to the recommendation of SwitchGear Genomics (Menlo Park, CA) with empty vector used as positive control for the transfection. The empty 3′UTR vector contains a constitutive promoter (found on all UTR constructs) and the luciferase gene (RenSP). This construct served as a positive control for the transfection. Cells were plated in 24-well plates and co-transfected with 5 nM of miR-195 or the negative control (Applied Biosystems), 0.12 µg of the vector (SwitchGear Genomics), and 0.75 µl of Lipofectamine (Invitrogen). The luminescence was read after 24 hours with the Light Switch assay system, using a SpectraMax M5e microplate reader.

### *In vivo* xenograft assay

Athymic nude female mice (five to six weeks old; body weight: 20–22 g) were obtained from the Frederick National Laboratory for Cancer Research animal facilities (Frederick, MD). After 48 hours of transfection with *ZNF367* siRNA and the negative control, three million cells were re-suspended in 100 µl of DMEM and Matrigel (1∶1), and they were injected into the flank of athymic nude mice.

### Statistical analyses

Continuous data is presented as mean ± standard deviation (SD) or standard error of mean (SEM). The Kruskal-Wallis test was used to compare nonparametric data from three or more groups. The Student's t-test or Mann-Whitney test was used to compare differences between two groups for parametric and nonparametric variables, respectively. The Pearson correlation test was used to identify correlations between two groups. A *p*-value < 0.05 was considered statistically significant. Statistical analysis was completed using Graph Pad Prism 5.0 statistical software.

## Results

### *ZNF367* is overexpressed in cancer

**Figure 1:**
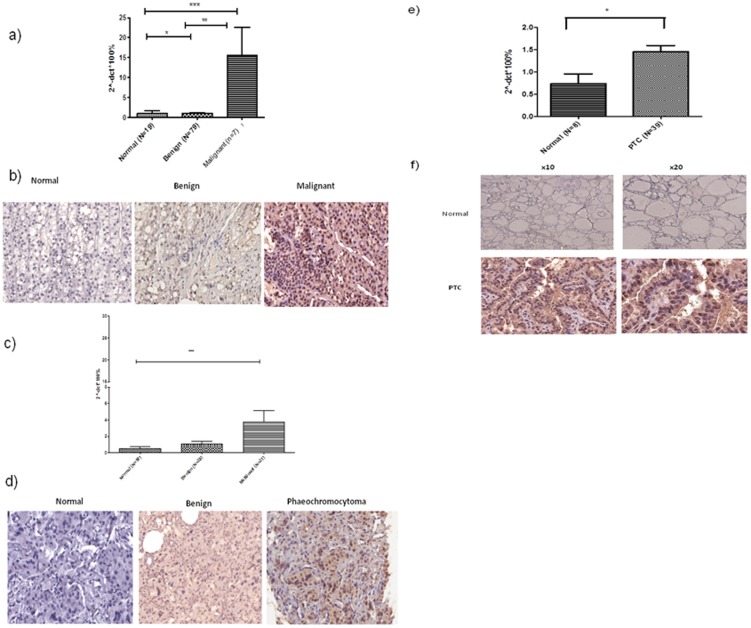
*ZNF367* mRNA and protein levels in adrenocortical carcinoma, papillary thyroid cancer, and pheochromocytoma/paraganglioma compared to benign and normal tissue samples for each cancer type. (A and B) Expression level in the normal adrenal cortex, benign adrenocortical adenomas, and adrenocortical carcinomas; (C and D) normal adrenal medulla, benign and malignant pheochromocytoma/paraganglioma tissue samples; and (E and F) normal thyroid and papillary thyroid cancer tissue samples. The Y axis on each graph represents the percentage of mRNA expression using the 2^∧-ΔCt^*100% method ± SEM. *p<0.05, **p<0.001, ***p<0.001 (Kruskal-Wallis test). Representative immunohistochemistry images are from normal, benign, and malignant tumor samples at 20X magnification.

*ZNF367* is overexpressed in adrenocortical carcinoma, papillary thyroid cancer, and malignant pheochromocytoma/paraganglioma, compared to benign and normal tissue samples for each tumor type (p<0.05; Figure 1). ZNF367 protein expression was present in the nucleus and cytoplasm. There was stronger ZNF367 staining in the nucleus than in the cytoplasm in each cancer sample (adrenocortical cancer, papillary thyroid cancer and malignant pheochromocytoma/paraganglioma). To confirm the elevated expression of *ZNF367* in adrenocortical carcinoma in a larger sample set, we analyzed expression profiling data from publicly available datasets deposited in gene expression omnibus. In two independent genome-wide gene expression data sets, *ZNF367* was overexpressed in adrenocortical carcinoma compared to adrenal cortical adenoma and normal tissue samples (p<0.001; Figure S1). These findings suggest that *ZNF367* is overexpressed in a variety of cancers, with consistent results across different analysis and genomic platforms used.

### *ZNF367* inhibits cellular proliferation, invasion, migration, and adhesion

Because *ZNF367* is overexpressed in endocrine cancer, we investigated its effect on cellular proliferation, invasion, and migration, events that are necessary for cancer progression. *ZNF367* was expressed in adrenocortical carcinoma (SW13, BD140A), papillary thyroid cancer (TPC-1), and HEK293 cell lines. Thus, we used both *ZNF367* knockdown and overexpression approaches to determine the effect of *ZNF367* on cellular proliferation, invasion, and migration. SiRNA knockdown achieved up to 80% knockdown of ZNF367 mRNA and protein expression compared to siRNA negative control (Figure S2).

**Figure 2:**
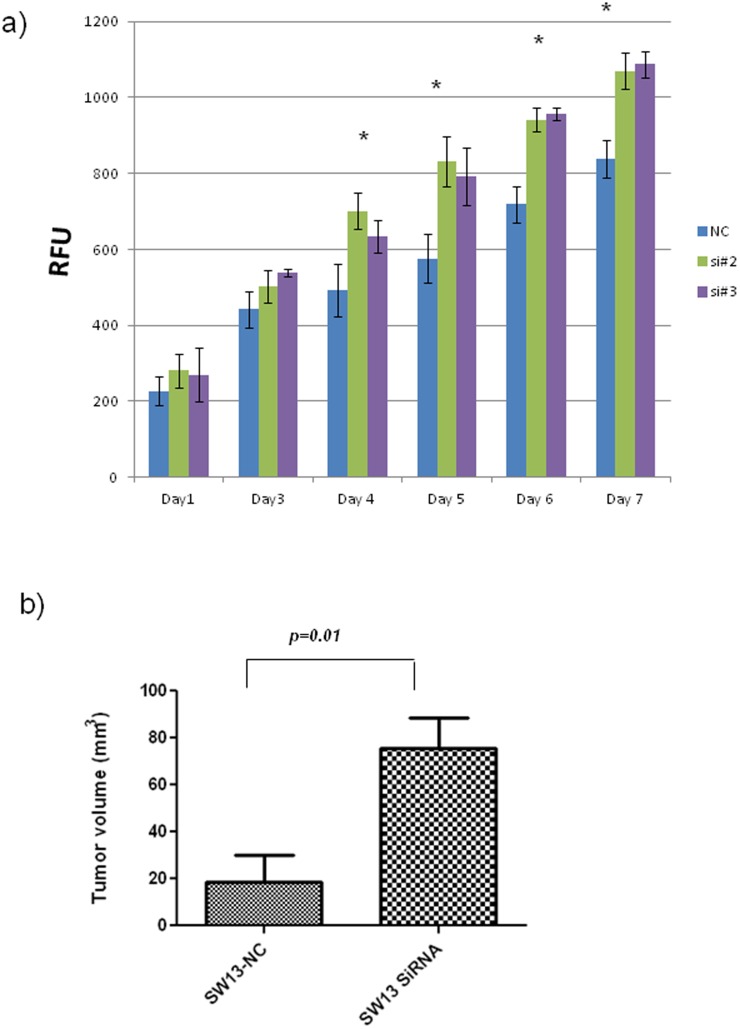
The effect of *ZNF367* knockdown on growth in SW13 cells in vitro and in vivo. (A) *ZNF367* knockdown increases cellular proliferation. The Y axis represents relative fluorescent units (RFU), and the X axis indicates days post-transfection. *p<0.05 relative to the negative control. Error bars represent ± SD. (B) *ZNF367* knockdown enhances tumor growth in vivo. SW13 cells were transfected with the negative control (n = 4) and siRNA (n = 4) into the right and left flank of each mouse. After 48 hours of transfection, 3×10^6^ cells were injected in athymic nude mice, and tumor growth was measured weekly. The Y axis represents the tumor volume and X axis the weeks of tumor measurement after flank injection. *p<0.05 and error bars represent ± SD.

**Figure 3:**
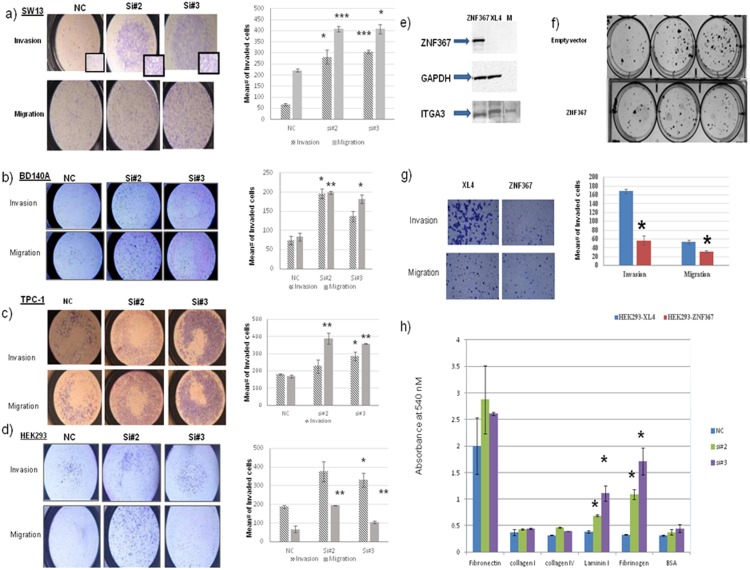
Effect of *ZNF367* knockdown and overexpression. *ZNF367* overexpression decreases cellular invasion and migration. (**A**) SW13, (**B**) BD104A, (**C**) TPC-1, and (**D**) HEK293 cell lines. After transfection, cells were plated inside a Boyden chamber for 48 hours. Cells were stained and counted in 4 fields. The left panel shows the representative image (12.5X) from the siRNA knockdown and negative control groups. The right panel indicates the quantitative measurement of invaded and migrated cells in knockdown and the negative control. *p<0.05 and error bars indicate ± SD. *ZNF367* overexpression decreases colony number, cellular invasion, and migration in HEK293 cells. (**E**) Western blot of *ZNF367* overexpression in HEK293 and SW13 cells (Empty vector, XL4). GAPDH was used as a loading control. (**F**) Representative clonogenic image for cells with ectopic *ZNF367* expression and its corresponding control (Empty vector, XL4). (**G**) Cellular invasion and migration decreased with *ZNF367* overexpression in HEK293 cells. The quantitative measurement of invaded and migrated cells per group (HEK293-HEK293-Empty vector or *ZNF367*) is represented in the bar graph on the right panel. (**H**) The extracellular protein attachment of SW13 cells with *ZNF367* knockdown. Cells were transfected with *ZNF367* siRNA and negative control siRNA, and were plated into adhesion plates and incubated for 90 minutes. The Y axis represents absorbance at 540 nM of cells adherent to each protein. Error bars represent ± SEM. *p<0.05, ***p<0.001.

*ZNF367* knockdown in SW13 cells, increased cellular proliferation (30–40%) *in vitro* and (3.5-fold) *in vivo* (p<0.05; Figure 2). *ZNF367* knockdown also increased cellular invasion and migration (p<0.05; Figure 3A). Given the dramatic effect of *ZNF367* on cellular invasion and migration in SW13 cells, the effect of *ZNF367* knockdown on cellular invasion and migration was also evaluated in BD140A, TPC-1, and HEK293 cell lines. The knockdown had a similar effect on the cellular invasion and migration of BD140A, TPC-1, and HEK293 cell lines (p<0.05; Figure 3, B–D). The effect of *ZNF367* on cellular growth, invasion, and migration was also confirmed by overexpressing *ZNF367* in HEK293 cells. *ZNF367* overexpression decreased cellular invasion, migration, and colony formation compared to the empty vector control (Figure 3E-G).

Because cellular invasion and migration require cell adhesion to the extracellular matrix, we evaluated the effect of *ZNF367* knockdown on cellular adhesion to extracellular matrix proteins. We found increased cellular adhesion, with *ZNF367* knockdown, to Laminin I (two–three-fold high) and fibrinogen (three–six-fold high) (p<0.05; Figure 3H). These proteins are known to function to enhance cellular attachment to the basement membrane or extracellular matrix through integrin receptors, which allow cancer cell adhesion and migration.

### *ZNF367* regulates ITGA3 expression

**Figure 4:**
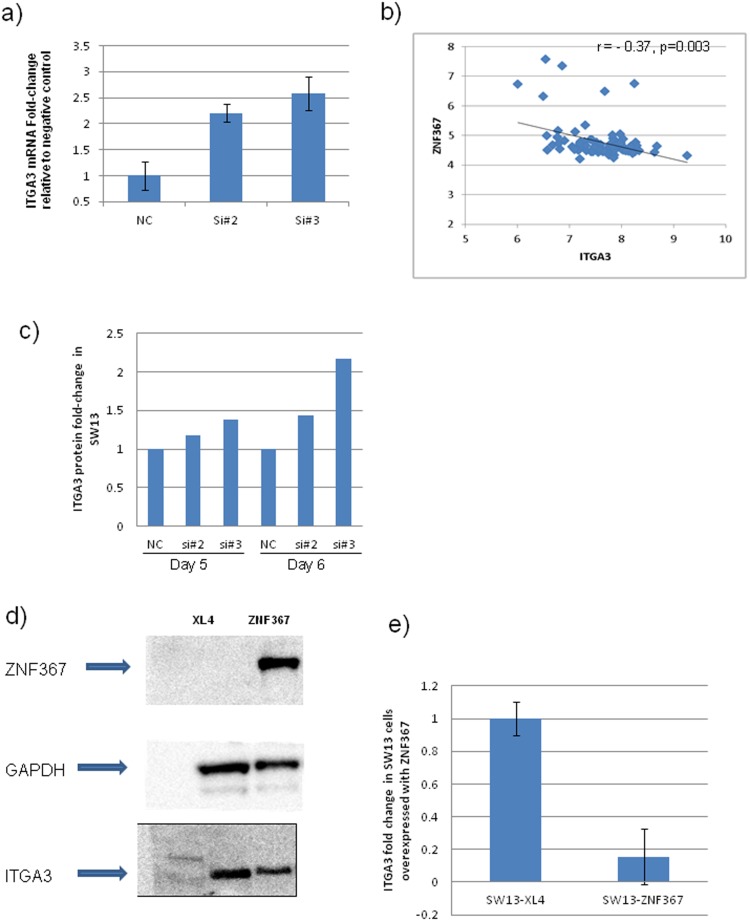
*ZNF367* regulates *ITGA3* expression. (**A**) *ZNF367* knockdown upregulates *ITGA3* expression in SW13 cells. Error bars represent ± SEM. (**B**) The correlation between *ITGA3* and *ZNF367* mRNA expression in adrenocortical tumor samples. X and Y axes represent log 2–transformed values. (**C**) Western blot quantification of ITGA3 protein expression with *ZNF367* knockdown. (**D**–**E**) ITGA3 expression with ZNF367 overexpression. Error bars represent ± SEM.

We were interested in identifying the gene(s) and pathway(s) regulated by *ZNF367*, given the effect of *ZNF367* on cellular invasion, migration, and adhesion, and because it is a transcription factor predicted to regulate gene expression. Using genome-wide mRNA expression analysis in cells transfected with *ZNF367* and negative control siRNAs, we identified two candidate genes (*ITGA3,* serpin peptidase inhibitor, clade B (ovalbumin), member 9 [*SERPINB9*]) possibly regulated by *ZNF367* based on applying several filter criteria (FDR<0.25, fold-change>1.5, common to all siRNA knockdown, and strong correlation in expression in human tumor samples) (Figure S3). *ITGA3* was chosen as a promising candidate for further study because it plays a significant role in cellular adhesion and invasion and was validated to be upregulated up to 2.5-fold with *ZNF367* knockdown (p<0.05; Figure 4A) [11]. Moreover, ITGA3 mRNA expression was inversely correlated with ZNF367 mRNA expression in human adrenocortical tumor samples (r = - 0.37, p = 0.015; Figure 4B). ITGA3 protein expression was also increased with siRNA knockdown of *ZNF367* (Figure 4C). On the other hand, overexpression of *ZNF367* reduced *ITGA3* expression compared to the empty vector (Figure 4D-E). These data suggest that *ZNF367* regulates cellular adhesion, invasion, and migration through its effect, at least in part, on *ITGA3* expression.

### MiR-195 directly targets *ZNF367* and regulates cellular invasion

**Figure 5:**
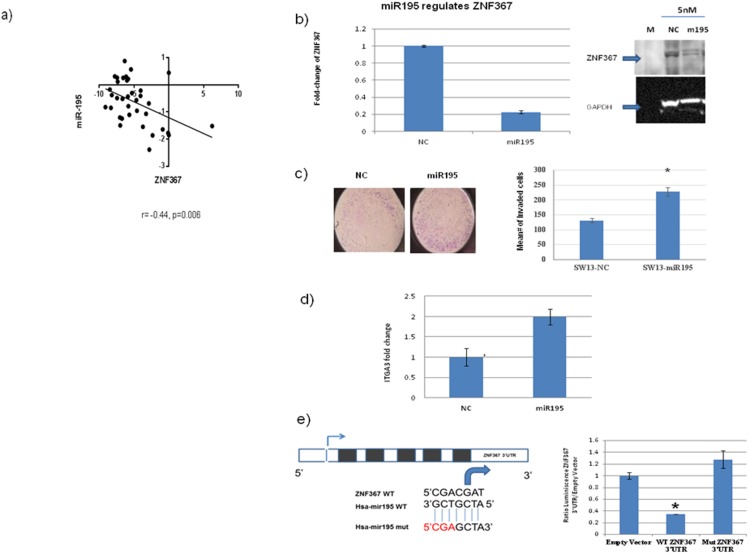
MiR-195 targets ZNF367. (**A**) The correlation between miR-195 and *ZNF367* expression in an adrenocortical tumor. The Pearson correlation coefficient is indicated by r with its p value. (**B**) Ectopic overexpression of pre-miR-195 results in downregulation of *ZNF367*. *ZNF367* mRNA expression in SW13 cells transfected with pre-miR-195 and the pre-negative control at 5 nM for 24 hours (presented as a fold-change relative to the pre-negative control). Error bars represent ± SEM. The right panel shows the Western blot of the ZNF367 protein from SW13 cells transfected with pre-miR-195 and the negative control. (**C**) Pre-miR-195 overexpression increases invasion in SW13 cells compared to the negative control. The right panel shows the mean number of invaded cells on the Y axis. (**D**) *ITGA3* mRNA expression is increased after transfection of miR-195 and the negative control in SW13 cells. Error bars represent ± SEM. (**E**) The binding site of miR-195 in the *ZNF367* 3′UTR, along with the mutant construct in the predicted seed region. In the right panel, the luciferase assay demonstrates decreased luminescence in SW13 cells co-transfected with miR-195 and the negative control at 5 nM, with the empty vector, wild-type *ZNF367* 3′UTR, or MUT-*ZNF367* 3′UTR vector (mutated in the first three nucleotides of the seed sequence). The luminescence was read after 24 hours of transfection. The Y axis represents the ratio of *ZNF367* 3′UTR to the empty vector. *p value < 0.05 compared to the negative control. Error bars represent ± SEM.

To understand the mechanism by which *ZNF367* expression is dysregulated in cancer, we postulated that microRNAs may be responsible for *ZNF367* overexpression. To identify candidate microRNAs that may target *ZNF367*, we queried the Target scan and miRDB databases for microRNAs predicted to target *ZNF367*. A total of 3 out of 14 microRNAs, predicted to target *ZNF367,* were found to be differentially expressed in adrenocortical carcinoma (Table S1). However, only miR-195 was significantly inversely correlated with *ZNF367* expression (r = -0.44; Figure 5A).

To investigate if miR-195 regulates *ZNF367* expression, cells were transfected with pre-miR-195 and pre-negative control (pre-NC), which showed reduced *ZNF367* mRNA and protein expression (Figure 5B). Moreover, overexpression of miR-195 showed increased *ITGA3* mRNA expression (two-fold) and cellular invasion compared to the negative control (p<0.05; Figure 5, C–D). To confirm that *ZNF367* is a direct target of miR-195, we performed luciferase assays to determine whether miR-195 binds to the 3′UTR of *ZNF367* mRNA. We observed significant reduced luciferase activity with miR-195 overexpression in wild-type 3′UTR compared to negative control and mutated *ZNF367* 3′UTR-transfected cells (p<0.01; Figure 5E). Thus, these observations suggest that miR-195 negatively regulates the expression of *ZNF367* by directly targeting its 3′UTR, resulting in decreased cellular invasion, the most dramatic effect of *ZNF367* that we observed in our functional studies.

## Discussion

To our knowledge, this is the first study to characterize the function of *ZNF367* in cancers. We found that *ZNF367* was overexpressed in a variety of endocrine cancers (adrenocortical carcinoma, papillary thyroid cancer, malignant pheochromocytoma/paraganglioma) compared to benign and or normal tissue samples. Functional *in vitro* and *in vivo* knockdown and overexpression studies demonstrated that *ZNF367* regulates cellular proliferation, invasion, migration, and adhesion. We used genome-wide gene expression analysis to identify candidate genes that are regulated by *ZNF367*, and we identified *ITAG3* as a gene that likely mediates the effect of *ZNF367* on cellular adhesion, invasion, and migration. Moreover, in silico analysis of human tumor sample genome-wide gene expression analysis demonstrated an inverse relationship between *ZNF367* and *ITAG3* expression. Lastly, we demonstrate that dysregulated *ZNF367* expression was associated with the loss of miR-195 expression in tumor samples and that this microRNA directly targets *ZNF367* and regulates cellular invasion, providing an understanding of the mechanism for dysregulated *ZNF367* expression. Based on these findings, we propose that there is a miR-195-*ZNF367-ITAG3* axis that regulates endocrine cancer progression.

This is the first study to characterize the expression and function of *ZNF367* in cancer. *ZNF367* knockout in mice has shown no significant abnormalities [12]. According to the Catalogue of Somatic Mutations in Cancer (COSMIC) database (http://cancer.sanger.ac.uk/cosmic/gene/overview?ln=ZNF367), missense, nonsense, and insertion mutations of *ZNF367* have been identified at a low rate (16 of 5,089 samples) in a variety of cancers. Nonetheless, using both knockdown and overexpression strategies, we found that *ZNF367* inhibited cancer growth and invasion, which suggests that the elevated levels in cancer samples are counter-regulatory and inhibit cancer progression. This finding is consistent with recent studies showing the diverse functions of other ZNF proteins in cancer biology and in mediating resistance to chemotherapy [9], [10], [13]-[15]. Moreover, we demonstrate that the increased levels of *ZNF367* are likely a result of a loss in expression of miR-195.

MiR-195 is a member of the miR-15 family of microRNAs that have been shown to have a role in cancer [16]. We and others have previously reported that miR-195 is downregulated in adrenocortical carcinoma and thyroid cancer, and that it is a prognostic marker in cancer [17]-[21]. Moreover, miR-195 has been shown to have a tumor suppressive function by regulating cell proliferation and apoptosis [22]-[24]. We identified the loss of miR-195 expression to be associated with increased *ZNF367* expression in human cancer samples based on integrated analysis of microRNA expression data and the target prediction database, and we demonstrated that miR-195 directly targets *ZNF367*. MiR-195 overexpression resulted in increased cellular invasion, recapitulating the effect of *ZNF367* on cellular invasion, and is consistent with its effect on cellular invasion in trophoblasts, in contrast to its effect on osteosarcoma and glioblastoma cells [22], [25], [26]. Such divergent effects of microRNAs on cellular invasion have previously been observed for other microRNAs, suggesting that miR-195 has a cell-specific effect. We propose that the loss of miR-195 expression results in the increased expression of *ZNF367* and a miR-195-*ZNF367* axis that is important in inhibiting cancer progression.

Given the significant effect of *ZNF367* on cellular adhesion, migration, and invasion, which are the necessary steps for cancer progression, we investigated the possible gene(s) that may inhibit this progression as a result of high levels of *ZNF367* in cancer, and we found that *ITGA3* expression is modulated by *ZNF367* and miR-195, and that it is inversely correlated in human tumor samples with *ZNF367* expression. *ITGA3* is a member of the integrin family of proteins, which function as a cell surface adhesion molecules and has a role in cancer invasion [27]. The increased *ITGA3* levels from *ZNF367* knockdown and increased adhesion of cells to Laminin I and Fibronectin suggest that decreased *ITGA3* may mediate the inhibitory effect of *ZNF367* on cellular adhesion and invasion. Thus, we propose a miR-195-*ZNF367-ITGA3* axis that regulates cancer progression.

In summary, *ZNF367* inhibits cancer progression by modulating cellular growth and invasion. *ZNF367* expression is directly regulated by miR-195, and the loss of miR-195 is associated with increased *ZNF367* in human cancer samples. The effect of *ZNF367* on cellular invasion, at least in part, may be mediated by *ITGA3*. Taken together, this data suggest that *ZNF367* regulates endocrine cancer progression.

## Supporting Information

Figure S1 ***ZNF367*** **mRNA expression from adrenocortical carcinoma genome-wide gene expression datasets.** (**A**) *ZNF367* mRNA expression levels from Giordano et al., 2009 (http://www.ebi.ac.uk/arrayexpress/experiments/E-GEOD-10927/), and (**B)** *ZNF367* mRNA expression levels from Reynies et al., 2009 (http://www.ebi.ac.uk/arrayexpress/experiments/E-TABM-311/). The Y axis on each graph represents Log 2–transformed values. *p<0.01, **p<0.001, ***p value<0.001.
(TIF)

Figure S2 ***ZNF367*** **knockdown in cell lines. (A) SW13 cells, (B) BD140A, (C) TPC-1, and (D) HEK293 cell lines**. Cells were transfected with *ZNF367* siRNA and the negative control (80 nM). The right panel represents protein levels that were determined after 5 days of transfection (NC indicates the negative control and si# indicates the specific siRNA targeting *ZNF367*). The Y axis of each graph indicates the *ZNF367* mRNA fold-change relative to the negative control.
(TIF)

Figure S3 **Algorithm for identifying candidate genes regulated by** ***ZNF367*** **.**
(TIF)

Table S1 Differentially expressed microRNAs in adrenocortical carcinoma that target ZNF367#.
(DOC)

## References

[1] Hanahan D, Weinberg RA (2011) Hallmarks of cancer: the next generation. Cell 144: 646–674. 21376230 10.1016/j.cell.2011.02.013

[2] Davies H, Bignell GR, Cox C, Stephens P, Edkins S, et al (2002) Mutations of the BRAF gene in human cancer. Nature 417: 949–954. 12068308 10.1038/nature00766

[3] Xing M, Alzahrani AS, Carson KA, Viola D, Elisei R, et al (2013) Association between BRAF V600E mutation and mortality in patients with papillary thyroid cancer. JAMA 309: 1493–1501. 23571588 10.1001/jama.2013.3190 PMC3791140

[4] Pasini B, Stratakis CA (2009) SDH mutations in tumorigenesis and inherited endocrine tumours: lesson from the phaeochromocytoma-paraganglioma syndromes. J Intern Med 266: 19–42. 19522823 10.1111/j.1365-2796.2009.02111.x PMC3163304

[5] Xekouki P, Pacak K, Almeida M, Wassif CA, Rustin P, et al (2012) Succinate dehydrogenase (SDH) D subunit (SDHD) inactivation in a growth-hormone-producing pituitary tumor: a new association for SDH? J Clin Endocrinol Metab 97: E357–366. 22170724 10.1210/jc.2011-1179 PMC3319210

[6] Asano H, Murate T, Naoe T, Saito H, Stamatoyannopoulos G (2004) Molecular cloning and characterization of ZFF29: a protein containing a unique Cys2His2 zinc-finger motif. Biochem J 384: 647–653. 15344908 10.1042/BJ20040394 PMC1134151

[7] Gilligan P, Brenner S, Venkatesh B (2002) Fugu and human sequence comparison identifies novel human genes and conserved non-coding sequences. Gene 294: 35–44. 12234665 10.1016/s0378-1119(02)00793-x

[8] Seyhan AA, Varadarajan U, Choe S, Liu W, Ryan TE (2012) A genome-wide RNAi screen identifies novel targets of neratinib resistance leading to identification of potential drug resistant genetic markers. Mol Biosyst 8: 1553–1570. 22446932 10.1039/c2mb05512k

[9] Wang T, Wang XG, Xu JH, Wu XP, Qiu HL, et al (2012) Overexpression of the human ZNF300 gene enhances growth and metastasis of cancer cells through activating NF-kB pathway. J Cell Mol Med 16: 1134–1145. 21777376 10.1111/j.1582-4934.2011.01388.x PMC4365892

[10] Duan Z, Choy E, Harmon D, Yang C, Ryu K, et al (2009) ZNF93 increases resistance to ET-743 (Trabectedin; Yondelis) and PM00104 (Zalypsis) in human cancer cell lines. PLoS One 4: e6967. 19742314 10.1371/journal.pone.0006967 PMC2734182

[11] Sachs N, Secades P, van Hulst L, Kreft M, Song JY, et al (2012) Loss of integrin alpha3 prevents skin tumor formation by promoting epidermal turnover and depletion of slow-cycling cells. Proc Natl Acad Sci U S A 109: 21468–21473. 23236172 10.1073/pnas.1204614110 PMC3535625

[12] Gerdin AK (2010) The Sanger Mouse Genetics Programme: high throughput characterisation of knockout mice. Acta Ophthalmologica 88.

[13] Rink L, Skorobogatko Y, Kossenkov AV, Belinsky MG, Pajak T, et al (2009) Gene expression signatures and response to imatinib mesylate in gastrointestinal stromal tumor. Mol Cancer Ther 8: 2172–2182. 19671739 10.1158/1535-7163.MCT-09-0193 PMC2822341

[14] Rossi M, Abbondanza C, D'Arcangelo A, Gazzerro P, Medici N, et al (2004) The Zn-finger domain of RIZ protein promotes MCF-7 cell proliferation. Cancer Lett 215: 229–237. 15488642 10.1016/j.canlet.2004.05.014

[15] Kuramoto K, Uesaka T, Kimura A, Kobayashi M, Watanabe H, et al (2000) ZK7, a novel zinc finger gene, is induced by vascular endothelial growth factor and inhibits apoptotic death in hematopoietic cells. Cancer Res 60: 425–430. 10667597

[16] Finnerty JR, Wang WX, Hebert SS, Wilfred BR, Mao G, et al (2010) The miR-15/107 group of microRNA genes: evolutionary biology, cellular functions, and roles in human diseases. J Mol Biol 402: 491–509. 20678503 10.1016/j.jmb.2010.07.051 PMC2978331

[17] Ozata DM, Caramuta S, Velazquez-Fernandez D, Akcakaya P, Xie H, et al (2011) The role of microRNA deregulation in the pathogenesis of adrenocortical carcinoma. Endocr Relat Cancer 18: 643–655. 21859927 10.1530/ERC-11-0082 PMC3201061

[18] Soon PS, Tacon LJ, Gill AJ, Bambach CP, Sywak MS, et al (2009) miR-195 and miR-483-5p Identified as Predictors of Poor Prognosis in Adrenocortical Cancer. Clin Cancer Res 15: 7684–7692. 19996210 10.1158/1078-0432.CCR-09-1587

[19] Akama T, Sue M, Kawashima A, Wu H, Tanigawa K, et al (2012) Identification of microRNAs that mediate thyroid cell growth induced by TSH. Mol Endocrinol 26: 493–501. 22301781 10.1210/me.2011-1004 PMC5417127

[20] Slaby O, Redova M, Poprach A, Nekvindova J, Iliev R, et al (2012) Identification of MicroRNAs associated with early relapse after nephrectomy in renal cell carcinoma patients. Genes Chromosomes Cancer 51: 707–716. 22492545 10.1002/gcc.21957

[21] Wang X, Wang J, Ma H, Zhang J, Zhou X (2012) Downregulation of miR-195 correlates with lymph node metastasis and poor prognosis in colorectal cancer. Med Oncol 29: 919–927. 21390519 10.1007/s12032-011-9880-5

[22] Zhang QQ, Xu H, Huang MB, Ma LM, Huang QJ, et al (2012) MicroRNA-195 plays a tumor-suppressor role in human glioblastoma cells by targeting signaling pathways involved in cellular proliferation and invasion. Neuro Oncol 14: 278–287. 22217655 10.1093/neuonc/nor216 PMC3280801

[23] Li D, Zhao Y, Liu C, Chen X, Qi Y, et al (2011) Analysis of MiR-195 and MiR-497 expression, regulation and role in breast cancer. Clin Cancer Res 17: 1722–1730. 21350001 10.1158/1078-0432.CCR-10-1800

[24] Furuta M, Kozaki K, Tanimoto K, Tanaka S, Arii S, et al (2013) The tumor-suppressive miR-497-195 cluster targets multiple cell-cycle regulators in hepatocellular carcinoma. PLoS One 8: e60155. 23544130 10.1371/journal.pone.0060155 PMC3609788

[25] Mao JH, Zhou RP, Peng AF, Liu ZL, Huang SH, et al (2012) microRNA-195 suppresses osteosarcoma cell invasion and migration in vitro by targeting FASN. Oncol Lett 4: 1125–1129. 23162665 10.3892/ol.2012.863 PMC3499598

[26] Bai Y, Yang W, Yang HX, Liao Q, Ye G, et al (2012) Downregulated miR-195 detected in preeclamptic placenta affects trophoblast cell invasion via modulating ActRIIA expression. PLoS One 7: e38875. 22723898 10.1371/journal.pone.0038875 PMC3378540

[27] Akiyama SK, Olden K, Yamada KM (1995) Fibronectin and integrins in invasion and metastasis. Cancer Metastasis Rev 14: 173–189. 8548867 10.1007/BF00690290

