# Supplementary figures and images for: ZNF367 Inhibits Cancer Progression and Is Targeted by miR-195
Source: PLoS One. 2014 Jul 21;9(7):e101423. doi: 10.1371/journal.pone.0101423 (PMC4105551; doi:10.1371/journal.pone.0101423)

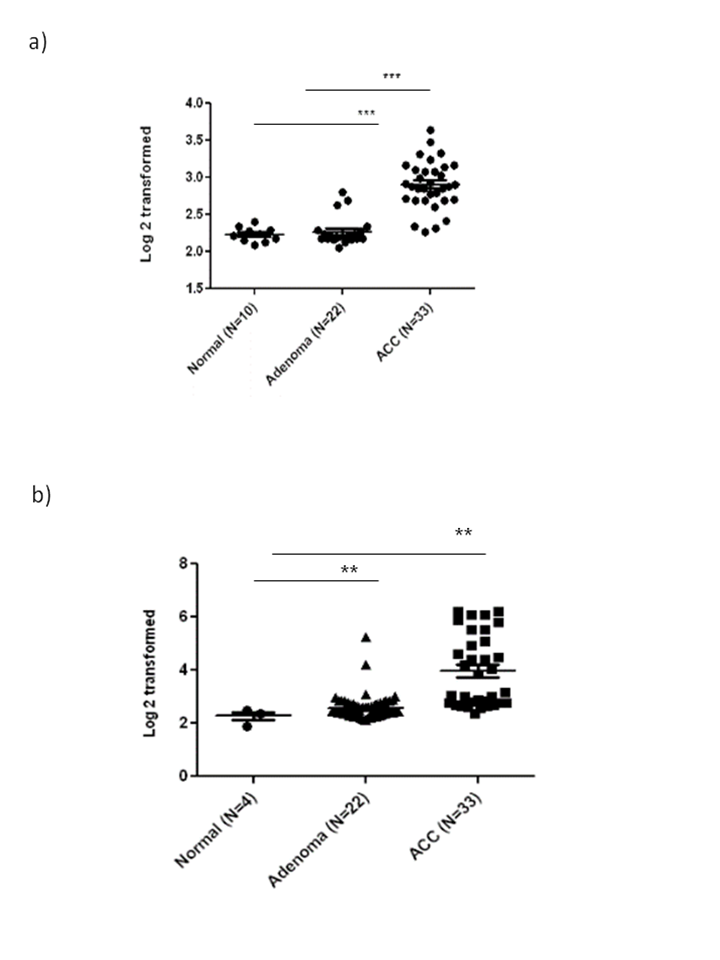

Supplement: Figure S1 — ZNF367 mRNA expression from adrenocortical carcinoma genome-wide gene expression datasets. (A) ZNF367 mRNA expression levels from Giordano et al., 2009 (http://www.ebi.ac.uk/arrayexpress/experiments/E-GEOD-10927/), and (B) ZNF367 mRNA expression levels from Reynies et al., 2009 (http://www.ebi.ac.uk/arrayexpress/experiments/E-TABM-311/). The Y axis on each graph represents Log 2–transformed values. *p<0.01, **p<0.001, ***p value<0.001. (TIF) [file pone.0101423.s001.tif]

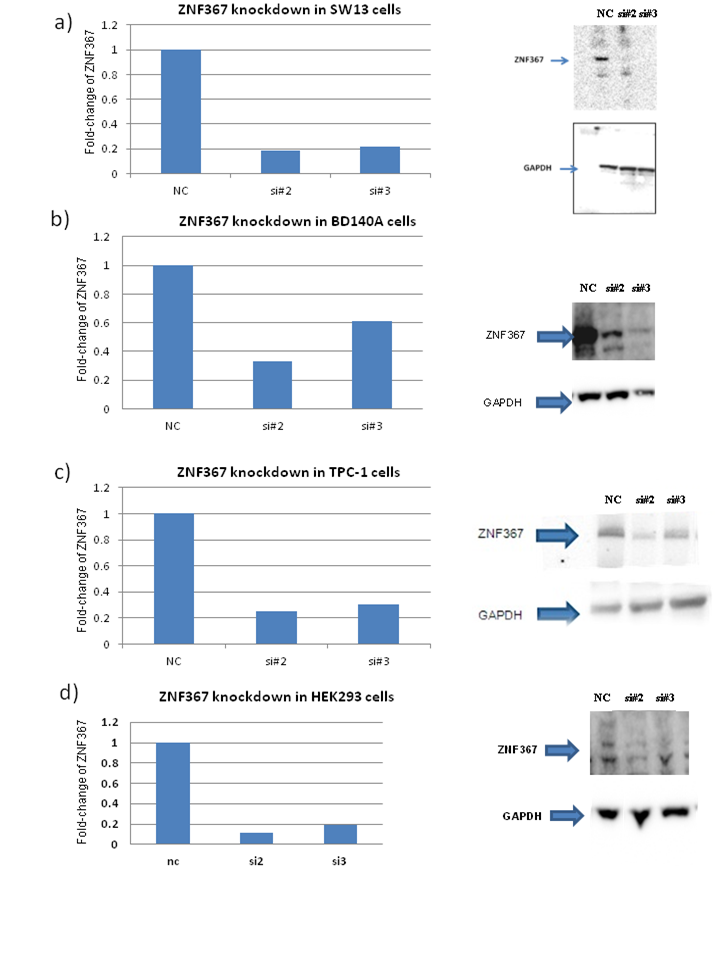

Supplement: Figure S2 — ZNF367 knockdown in cell lines. (A) SW13 cells, (B) BD140A, (C) TPC-1, and (D) HEK293 cell lines. Cells were transfected with ZNF367 siRNA and the negative control (80 nM). The right panel represents protein levels that were determined after 5 days of transfection (NC indicates the negative control and si# indicates the specific siRNA targeting ZNF367). The Y axis of each graph indicates the ZNF367 mRNA fold-change relative to the negative control. (TIF) [file pone.0101423.s002.tif]

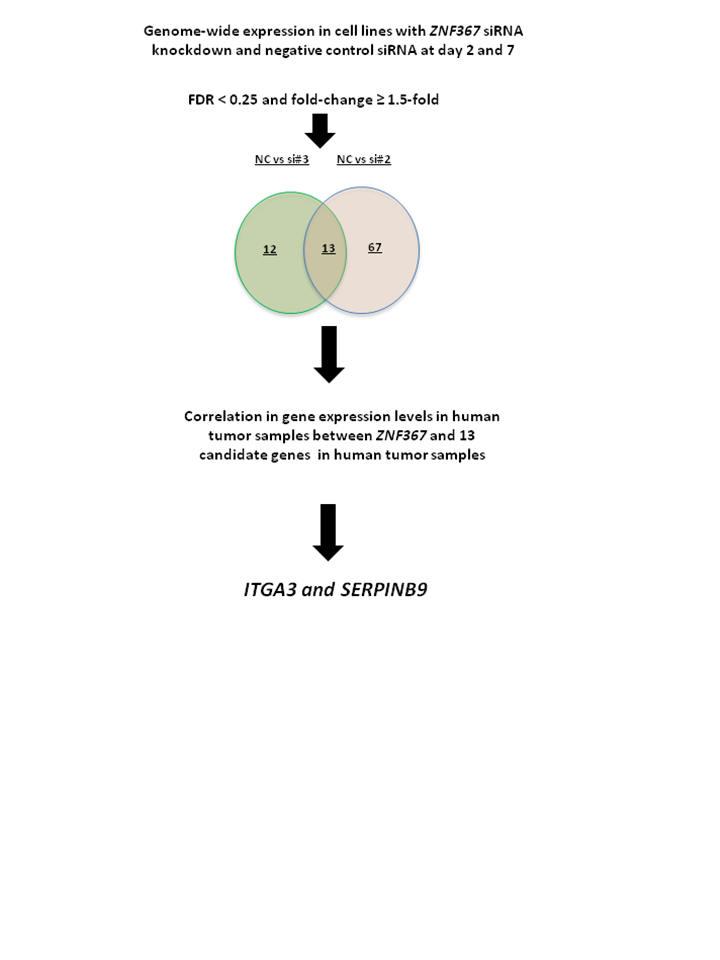

Supplement: Figure S3 — Algorithm for identifying candidate genes regulated by ZNF367 . (TIF) [file pone.0101423.s003.tif]
