# Supplementary material for: ZNF367 Inhibits Cancer Progression and Is Targeted by miR-195
Source: PLoS One. 2014 Jul 21;9(7):e101423. doi: 10.1371/journal.pone.0101423 (PMC4105551; doi:10.1371/journal.pone.0101423)
Supplement: Table S1 — Differentially expressed microRNAs in adrenocortical carcinoma that target ZNF367#. (DOC) [file pone.0101423.s004.doc]

**Table S1**. Differentially expressed microRNAs in adrenocortical carcinoma that target *ZNF367^#^*

| **microRNAs** | **Fold-change (malignant/benign)*** | **FDR^** |
| --- | --- | --- |
| hsa-miR-195 | -1.08 | 0.00074 |
| hsa-miR-664 | -0.41 | 0.0032 |
| hsa-miR-16 | -0.98 | 0.067 |

*^#^*Listed from lowest to highest false discovery rate.

*Fold-change log_2_

^FDR: false discovery rate
